# Supplementary material for: Can working in agriculture have a favorable effect on depressive symptoms? Life satisfaction as a mediator
Source: PLoS One. 2025 Aug 13;20(8):e0325912. doi: 10.1371/journal.pone.0325912 (PMC12349095; doi:10.1371/journal.pone.0325912)
Supplement: Attachment — Submitted filename: Reply to editor, reviewer1, reviewer2 and reviewer3.docx [file pone.0325912.s002.docx]

Journal requirements:

1. When submitting your revision, we need you to address these additional requirements.

Please ensure that your manuscript meets PLOS ONE's style requirements, including those for file naming. The PLOS ONE style templates can be found at 
https://journals.plos.org/plosone/s/file?id=wjVg/PLOSOne_formatting_sample_main_body.pdf and 
https://journals.plos.org/plosone/s/file?id=ba62/PLOSOne_formatting_sample_title_authors_affiliations.pdf

2. Thank you for stating in your Funding Statement: 
Funding
This research is supported by Key Laboratory of Philosophy and Social Sciences of Colleges and Universities in Guangdong Province (2023WSYS005, 2023WSYS007), the 13th Five-Year Plan of Guangdong Province for Philosophy and Social Sciences (GD20XGL42).

Please provide an amended statement that declares *all* the funding or sources of support (whether external or internal to your organization) received during this study, as detailed online in our guide for authors at http://journals.plos.org/plosone/s/submit-now.  Please also include the statement “There was no additional external funding received for this study.” in your updated Funding Statement. 
Please include your amended Funding Statement within your cover letter. We will change the online submission form on your behalf.

3. In the online submission form, you indicated that your data is available only on request from a third party. Please note that your Data Availability Statement is currently missing the name of the third party contact or institution. Please update your statement with the missing information.

4. PLOS requires an ORCID iD for the corresponding author in Editorial Manager on papers submitted after December 6th, 2016. Please ensure that you have an ORCID iD and that it is validated in Editorial Manager. To do this, go to ‘Update my Information’ (in the upper left-hand corner of the main menu), and click on the Fetch/Validate link next to the ORCID field. This will take you to the ORCID site and allow you to create a new iD or authenticate a pre-existing iD in Editorial Manager.

5. Your ethics statement should only appear in the Methods section of your manuscript. If your ethics statement is written in any section besides the Methods, please delete it from any other section.

 Dear Editor，

Thank you for your kind comment and rapid arrangement on our revision, we have carefully read and replied to every comment made by 3 reviewers on our manuscript. Please review our revised manuscript and response letter.

Hope everything smooth.

Best Regards,

Yuan Yao Chen

yuanyaochen0401@163.com

Reviewers' comments:

Reviewer's Responses to Questions

**Comments to the Author**

1. Is the manuscript technically sound, and do the data support the conclusions?

The manuscript must describe a technically sound piece of scientific research with data that supports the conclusions. Experiments must have been conducted rigorously, with appropriate controls, replication, and sample sizes. The conclusions must be drawn appropriately based on the data presented.

Reviewer #1: Yes

Reviewer #2: Yes

Reviewer #3: Yes

2. Has the statistical analysis been performed appropriately and rigorously?

Reviewer #1: Yes

Reviewer #2: Yes

Reviewer #3: Yes

3. Have the authors made all data underlying the findings in their manuscript fully available?

The [PLOS Data policy](http://www.plosone.org/static/policies.action#sharing) requires authors to make all data underlying the findings described in their manuscript fully available without restriction, with rare exception (please refer to the Data Availability Statement in the manuscript PDF file). The data should be provided as part of the manuscript or its supporting information, or deposited to a public repository. For example, in addition to summary statistics, the data points behind means, medians and variance measures should be available. If there are restrictions on publicly sharing data—e.g. participant privacy or use of data from a third party—those must be specified.

Reviewer #1: Yes

Reviewer #2: Yes

Reviewer #3: Yes

4. Is the manuscript presented in an intelligible fashion and written in standard English?

PLOS ONE does not copyedit accepted manuscripts, so the language in submitted articles must be clear, correct, and unambiguous. Any typographical or grammatical errors should be corrected at revision, so please note any specific errors here.

Reviewer #1: Yes

Reviewer #2: Yes

Reviewer #3: Yes

5. Review Comments to the Author

Please use the space provided to explain your answers to the questions above. You may also include additional comments for the author, including concerns about dual publication, research ethics, or publication ethics. (Please upload your review as an attachment if it exceeds 20,000 characters)

Dear Reviewer 1,

Thank you very much for your comments. I have read your comments carefully and made a modification to the original manuscript. The revised part was marked in yellow based on your comments. The following are my responses to your questions.

**Reviewer #1:** 1. Add 'China' to the keywords to improve the searches of the article when published on online databases.

Our response: Thank you for your kind comment and advise, we have revised and added the key word “China” into our key words.(It can be reviewed in the Key Words part in our revised manuscript in yellow):

“**Key Words**: Agriculture work; Depressive symptoms; Life satisfaction; CHARLS; China”

2. In the introduction, it is important to expose the research gaps by reviewing related studies in the Chinese context especially. While the global stretch of this have been done, only a study referencing China has been cited though there are a lot of studies in China that have investigated the correlation between job(s) and depression with life satisfaction playing a mediating role.

Our response: Thank you for your valuable feedback. Our research was conducted within the context of China, and it was an oversight on our part not to cite more studies specific to China. We have now included several references that examine the relationship between work and depressive symptoms within the Chinese context, highlighting the mediating role of life satisfaction,as follows(At Line 62-64 in revised manuscript):

“Research has shown that work is closely related to the development of depression[4], which can be subdivided into work pressure[5], work hours[6, 7] , work environment[8, 9],work support[10],subjective social status[11]and so on,...”

3. It's important to reference some of the studies that have validated the CES-D scale with much success, pointing to it as reliable and as such efficacious for such a study.

Our response: Thank you for your valuable comment, it is our flaw to make you confuse about our validity of the CES-D scale. We have cited previous researches about the scale to evident its validity and reliability, which is as follows(At Line 126 in revised manuscript):

“... ,which is now a widely used and proven valid and reliable common indicator of depression[19, 20].”

4. In the selection of the covariates, do well to reference the previous studies that were consulted and justify the covariates that were settled on for the study.

Our response: Thank you for your valuable comment.We have cited previous studies to demonstrate the relationships between each covariate and the dependent variable (depressive symptoms), thereby supporting the rationale for our choice of covariates.

5. In the discussion, the proposition put forward which is based on the significant results attained after running rigorous analysis, which are lower life satisfaction and social activity participation as major risk factors for the development of depression among agricultural workers need to be discussed from the perspective of other empirical studies. Which existing studies on work-related depression have recorded similar results? What might probably be the underlying causative factors for recording such results? Discussion of these questions must be expanded in the discussion section. This was beautifully done in the discussion on the high risk of depression recorded among self-employed agricultural workers.

Our response: Thank you for your valuable comment.We have expanded our discussion and thoroughly restructured the logical flow in **Discussion** section,as follows(At Line 329-344 in revised manuscript):

“The reasons for low life satisfaction among agricultural workers are complex, primarily influenced by the following factors:(1) Economic Pressure[41]: Agricultural workers’ incomes are generally less stable compared to those in non-agricultural occupations, as they are highly susceptible to weather changes, market fluctuations, and other external factors. The seasonal and uncertain nature of agricultural income can impose substantial financial stress, leading to lower life satisfaction.(2) Occupational Health Risks[42]: Agricultural workers face high occupational health risks, including long working hours, exposure to adverse weather conditions, and pesticide exposure mentioned earlier. These health risks negatively impact both physical and mental well-being, reducing overall life satisfaction.(3) Social Isolation and Lack of Social Support[41]: Agricultural workers are often geographically isolated due to the nature of their work, resulting in limited social interactions and relationships. This isolation contributes to lower levels of social participation. The lack of social support and limited opportunities for interaction may lead to feelings of loneliness and depressive symptoms, further impacting life satisfaction.(4) Mental Health Issues and Cultural Stigma[42]: In rural areas, mental health issues are still subject to cultural stigma, which makes it difficult for agricultural workers experiencing mental health challenges to seek help. The lack of mental health support resources can exacerbate these issues, leading to further declines in life satisfaction.”

6. The limitations must be moved from the discussion to the concluding section. Also, after summarizing the study's purpose, it is important to reiterate the key results and draw valid conclusions from them based on the underlying research questions and hypotheses set. This has not been done.

Our response：Thank you for your valuable advise and comment, we have adjusted the narration order, revised the language and moved it to the conclusion section. For helping you better review our revised part, the revised part is at conclusion section Line 413-420 of revised manuscript.

Best Regards,

Yuan Yao Chen

[yuanyaochen0401@163.com](mailto:yuanyaochen0401@163.com)

Dear Reviewer 2,

Thank you very much for your comments and appreciation. We have read your comments carefully and made a modification to the original manuscript. The revised part was marked in yellow based on your comments. The following are my responses to your questions.

**Reviewer #2:** The study “Can working in agriculture have a favorable effect on depressive symptoms? Life satisfaction as a mediator” investigates the impact of agricultural work on depressive symptoms and whether life satisfaction mediates this relationship. The study concluded that self-employed workers in agriculture have a higher risk of developing depressive states and present a high risk of developing depression. This is a relevant finding for research related to depressive disorders. However, there are areas that require clarification and significant improvements, particularly regarding the background, research design development, data analysis, and alignment of the research questions with the results. Addressing these points would strengthen the overall impact and credibility of the work. Notably, the manuscript is generally well-written, and the authors should be able to address my points through a revision. My comments and suggestions for improvement are described below.

Our response：Thank you for your appreciation, valuable comments and suggestions on our research, we have carefully read and responded to your comments as following responses. We have fixed and revised most of the problems you mentioned and still tried to improve our future study based your comments.

**Introduction**

1.2nd paragraph:
In the excerpt: "...but there is still no research focusing on the association between job type and depression…"
There is an inconsistency because the two references below associate depression and job type:

Our response：Inappropriate wording in our article was an oversight on our part. Our paper examines the relationship between two types of jobs—agricultural and non-agricultural work which we have made an explanation at **Method**—and depressive symptoms. However, the definitions of “job type” in the two references below differ from ours. We are very grateful for your feedback on this matter and have made the following revisions accordingly (At Line 65 in revised manuscript):

“but there is still no research focusing on the association between job type—agricultural and non-agricultural— and depression.”

2.“This study will then look at different types of work - agricultural and non-agricultural - and explore the association with depression, and further analyze subgroups of the population according to whether they are self-employed or employed".
“Therefore, in this study, it is necessary to investigate whether the type of jobs has a correlation with life satisfaction and to include life satisfaction in the model as a mediating variable to investigate whether the type of jobs acts on the depressive state through life satisfaction”.I suggest focusing the research objectives in a single paragraph, preferably in the last paragraph of the Introduction.

3nd paragraph: In the excerpt: " ...It has been proven that there is an association between life satisfaction and depression in middle-aged and elderly people."
It is not clear what type of association exists between the two mentioned variables.

Our response：Thank you for your valuable comment.

(1)We have made revisions and consolidated the study objectives in the final paragraph of the introduction, as follows(At Line 79-83 in revised manuscript):
“This study will first examine the association between different types of work and depressive symptoms. Further, it will analyze population subgroups based on work state—self-employed versus employed—in order to provide more targeted public health recommendations. Additionally, life satisfaction will be incorporated into the model as a mediating variable to explore whether job types influence depressive symptoms through the pathway of life satisfaction.”
(2)The two referenced studies[14,15] reveal that previous research has established a correlation between depressive symptoms and life satisfaction. Reference [14] indicates that life satisfaction can serve as a potential indicator of depression, although it may be influenced by gender differences. Reference [15] highlights a reciprocal and enduring relationship between life satisfaction and depression.


**Method**
2.Research design
I suggest specifying the method used for the sample calculation.

Our response：Thank you for your valuable comment, it is our flaw that the method used for the sample calculation is not explained enough. Firstly, the data we use for analysis in this article comes from the public database CHARLS,which are covered in detail in the **Method_ Research design** section.For the convenience of your review, we have modified the flow chart (Fig.1) to make you more clear about the method used in our sample calculation. Additionally,We have added our methodology for data analysis as follows(At Line 173 in revised manuscript):

“The above statistical analysis is based on R-4.3.1.”

3.Study population
I suggest reporting the exclusion criteria applied to the study.

Our response：Thank you for your valuable comment.In our study, the criteria for inclusion of subjects at baseline were: subjects who contained a record of jobs type , assessment of depressive states, assessment of life satisfaction, age, gender, type of residence, work states, marital states, socially engaged activity score, smoking states, drinking states, sleep duration, prior work states in CHARLS 2015. Meanwhile, we have excluded subjects that have missing values in the variables given upon.We have explained this in the **Methods_Study population** section of the manuscript.For the convenience of your review, we have modified the flow chart (Fig.1) to make you more clear about our exclusion criteria.

4.Measurement Tools
Life satisfaction
In the excerpt: “Life satisfaction was assessed at baseline by asking participants: How satisfied are you with your life in general?"
Did the participants receive any information about what life satisfaction is and/or some more objective criteria to assist in their self-analysis?
I believe that a subjective question may lead participants to potential interpretation biases, making it necessary to establish criteria for them to respond more accurately.

Our response：Thank you for your valuable comment.The data we use for analysis in this article comes from the public database CHARLS.There is a question "How satisfied are you with your life in general?" included in the questionnaire of CHARLS,which have been proved that strongly correlates with the validated 5-item Satisfaction with Life Scale (SWLS).We have cited the previous research[21] to explain this correlation.

5.Methods of statistical analysis
It is not clear what covariates were included in the correlation analysis.

Our response：Thank you for your valuable comment.The covariates included in our study cover demographic characteristics such as age, gender, type of residential address and marital states, as well as factors that have been validated by research to be highly correlated with depressive states, such as social activity participation, smoking states, drinking states.We have illustrated in **Method_Selection of covariates** and shown in Fig.3.

**Result/Discussion**
6.I suggest reporting the influence of working hours and its relationship with depression and life satisfaction of agricultural workers.

Our response：Thank you for your valuable comment.Certainly, as you mentioned, this is a very worthwhile area of study. However, because we used a public database, the questionnaire design in this database did not include questions regarding ‘working hours.’ Consequently, we lack a data source on working hours, which limits our ability in this study to explore the effects of working hours and their relationship with depressive symptoms and life satisfaction among agricultural workers. We are eager and will strive to incorporate this direction into future research as we undertake independent studies.

**Discussion**
7.4nd paragraph:
In the excerpt: “There are many factors that contribute to the high prevalence of depressive states in the female population, including a combination of biological, psychological, and sociocultural factors. Biologically, women are more sensitive to hormonal fluctuations associated with the reproductive cycle, such as hormonal fluctuations (menstrual cycle, postpartum, and menopause) that can affect mood thus leading to the occurrence of premenstrual irritability disorder and postpartum depressive states, among others[32 –34]; psychology points out that women are more prone to recurring thoughts of negativity[32], which may be associated with an increased risk of depressive states; and in the process of growing up, the Gender roles and societal expectations place additional stress on women, with sexual violence [35], sexual abuse [33], interpersonal violence, stress exposure and stress susceptibility [33] at the micro level, and gender inequality[36, 37] at the macro level, all of which increase the risk for the development of depressive states in women.”
What is the relationship between the mentioned factors and depression associated with agricultural work?

Our response: Thank you for your comment and valuable question, it is our flaw not to narrative clearly about the association between Agricultural labor related depression and female factor. We have already added extended discussion and fully reorganized our logic in the discussion part. To help you better review our revised part, it i as follows:

“The reasons for low life satisfaction among agricultural workers are complex, primarily influenced by the following factors:(1) Economic Pressure[41]: Agricultural workers’ incomes are generally less stable compared to those in non-agricultural occupations, as they are highly susceptible to weather changes, market fluctuations, and other external factors. The seasonal and uncertain nature of agricultural income can impose substantial financial stress, leading to lower life satisfaction.(2) Occupational Health Risks[42]: Agricultural workers face high occupational health risks, including long working hours, exposure to adverse weather conditions, and pesticide exposure mentioned earlier. These health risks negatively impact both physical and mental well-being, reducing overall life satisfaction.(3) Social Isolation and Lack of Social Support[41]: Agricultural workers are often geographically isolated due to the nature of their work, resulting in limited social interactions and relationships. This isolation contributes to lower levels of social participation. The lack of social support and limited opportunities for interaction may lead to feelings of loneliness and depressive symptoms, further impacting life satisfaction.(4) Mental Health Issues and Cultural Stigma[42]: In rural areas, mental health issues are still subject to cultural stigma, which makes it difficult for agricultural workers experiencing mental health challenges to seek help. The lack of mental health support resources can exacerbate these issues, leading to further declines in life satisfaction.”

**Conclusion**
8.I suggest reporting the relevance of the research for public health policies in China. In light of the study's results, what can be done to improve the quality of life for agricultural workers?

Our response：Thank you for your precious suggestions, we have added suggestions about the public health policies in China to help improve the life satisfaction and well-being of agricultural workers in the Paragraph 5 of discussion section. It is as follows:

“For the agricultural self-employed and non-agricultural employed people, there are several suggestions we proposed : 1) Improve life satisfaction by improving the quality of life[43, 44]; 2) Participate in more social activities to enhance the interaction with friends and social support[21, 22, 28]; 3) Extend the sleep time and develop good sleep habits to get sufficient physical and mental rest and relaxation[45–47]. Moreover, for the government to complement public health policies for agricultural workers, we proposed following suggestions: 1) Construct more rural fitness and recreational facilities; 2) Conduct more mental health screening activities in rural areas; 3) Implement policies to improve the incomes of agricultural workers in rural areas.”

Best Regards,

Yuan Yao Chen

[yuanyaochen0401@163.com](mailto:yuanyaochen0401@163.com)

Dear Reviewer 3,

Thank you very much for your comments and approval on our manuscript! We have read your comments carefully and made a modification to the original manuscript. The revised part was marked in yellow based on your comments. The following are my responses to your comments.

**Reviewer #3:** 1. The study addresses an important and understudied area: the relationship between agricultural work and depressive symptoms. However, the use of a single-item measure for life satisfaction, while common, may not capture the full complexity of the construct. The study doesn't fully explore potential mechanisms underlying the relationship between agricultural work and depressive symptoms.

Our response: Thank you for your valuable comment.We have expanded our discussion and thoroughly restructured the logical flow in **Discussion** section,as follows(At Line 329-344 in revised manuscript):

“The reasons for low life satisfaction among agricultural workers are complex, primarily influenced by the following factors:(1) Economic Pressure[41]: Agricultural workers’ incomes are generally less stable compared to those in non-agricultural occupations, as they are highly susceptible to weather changes, market fluctuations, and other external factors. The seasonal and uncertain nature of agricultural income can impose substantial financial stress, leading to lower life satisfaction.(2) Occupational Health Risks[42]: Agricultural workers face high occupational health risks, including long working hours, exposure to adverse weather conditions, and pesticide exposure mentioned earlier. These health risks negatively impact both physical and mental well-being, reducing overall life satisfaction.(3) Social Isolation and Lack of Social Support[41]: Agricultural workers are often geographically isolated due to the nature of their work, resulting in limited social interactions and relationships. This isolation contributes to lower levels of social participation. The lack of social support and limited opportunities for interaction may lead to feelings of loneliness and depressive symptoms, further impacting life satisfaction.(4) Mental Health Issues and Cultural Stigma[42]: In rural areas, mental health issues are still subject to cultural stigma, which makes it difficult for agricultural workers experiencing mental health challenges to seek help. The lack of mental health support resources can exacerbate these issues, leading to further declines in life satisfaction.”

2. the article presents a well-structured study with a solid methodology and employing appropriate statistical techniques for the research questions. The use of propensity score matching to balance the agricultural and non-agricultural groups is a strength. The results are computed using appropriate statistical techniques, including logistic regression analysis to identify protective factors associated with life satisfaction and depressive symptoms. The use of odds ratios (OR) with 95% confidence intervals (CI) provides a clear understanding of the strength and significance of the associations. Additionally, mediation analysis is conducted to explore the role of life satisfaction in the relationship between job type and depressive symptoms, which is a robust approach to understanding causal pathways. The sensitivity analysis adds another layer of reliability to the findings, indicating that the mediating effects are not overly influenced by confounding variables. Overall, the statistical methods employed are appropriate for the research questions posed. However, there are some potential drawbacks:
• The dichotomization of the CES-D scores for logistic regression may lead to loss of information.
• The study doesn't address potential selection bias - people with certain characteristics may be more likely to engage in agricultural work.

Our response：Thank you for your approval on our study and hard work, we really appreciate it. For your valuable comments, first of all, using dichotomization on CES-D scores for logistic regression analysis had met our purpose of our study, which is to find out the complex associations between agricultural work and depression states through the mediation of life satisfaction. Moreover, for the potential selection bias, it is indeed our flaw not to consider it during the study process. However, due to the questionnaire structure of the open database we used didn’t have such inquiry, we will consider to improve and reduce the bias in the near future study. Thanks for your great advice again!

3. The finding that agricultural workers have higher depressive symptom scores is surprising and doesn't align with some existing literature on the potential mental health benefits of nature-based activities. However, this could be due to other factors associated with agricultural work in China, such as economic stress or physical strain. The mediating role of life satisfaction is consistent with previous research on well-being and mental health. The subgroup analyses provide interesting insights into how the relationships differ across various populations, which adds nuance to the overall findings.

Our response：We really appreciated your highly approbation on our research, which is our honor and pleasure. We will continue to dig out more interesting and valuable study of public health and occupational health in the near future. Your praise is our energy to keep going on our hard work.

4. There are numerous grammatical and typographical errors throughout the text, which should be carefully edited.

Our response：We really appreciated your careful review on our manuscript! We have already done revision on such grammatical and typographical errors in our revised manuscript.

Best Regards,

Yuan Yao Chen

[yuanyaochen0401@163.com](mailto:yuanyaochen0401@163.com)
