# Supplementary material for: Can working in agriculture have a favorable effect on depressive symptoms? Life satisfaction as a mediator
Source: PLoS One. 2025 Aug 13;20(8):e0325912. doi: 10.1371/journal.pone.0325912 (PMC12349095; doi:10.1371/journal.pone.0325912)
Supplement: Attachment — Submitted filename: revised manuscript 3.0.docx [file pone.0325912.s003.docx]

**Can working in agriculture have a favorable effect on depressive symptoms?Life satisfaction as a mediator**

Feiying He^1†^, Xiaoying Li^2†^ ,Xiangchun Xu^3†^, Shulin Bao^2^, Hualin Liu^4,5^, Yanwu Chen^4,5^, Yuan Yao Chen^4,5*^

^1^School of Health Management, Southern Medical University, Guangzhou, China

^2^School of Basic Medicine, Southern Medical University, Guangzhou, 510515, China

^3^Nanfang Hospital, Southern Medical University, Guangzhou, 510515, China

^4^Research & Development Division, Perfect Life & Health Institute, Zhongshan, China.

^5^Research & Development Division, Perfect (Guangdong) Co., Ltd., Zhongshan, China.

† Feiying He, Xiaoying Li ,and Xiangchun Xu contributed equally to this work and share first authorship.

* Corresponding authors:

Yuan Yao Chen: Research & Development Division, Perfect Life & Health Institute, Zhongshan, China.& Research & Development Division, Perfect (Guangdong) Co., Ltd., Zhongshan, China.

Email:yuanyaochen0401@163.com;

ORCID:

Feiying He: 0000-0001-6621-0279

Xiaoying Li: 0009-0005-4244-6865

Xiangchun Xu:0009-0004-1513-9811

Shulin Bao:0009-0008-7997-5973

Yuan Yao Chen:0000-0003-2962-3466

Yanwu Chen:0009-0004-1583-5104

Hualin Liu:0000-0002-3630-5522

#### Abstract

**Purpose**

Several studies have explored the relationship between various aspects of work and the onset of depressive symptoms. However, there is a lack of research focusing on the association between job types and depressive symptoms. This study aims to investigate the impact of agricultural work on depressive symptoms and whether life satisfaction mediates this relationship.

**Methods**

Data were obtained from the 2015 China Health and Retirement Longitudinal Study (CHARLS) (n = 6856). Participants were categorized based on whether they were engaged in agricultural or non-agricultural work, and further classified as self-employed or employed. Depressive symptoms and life satisfaction were assessed using the CES-D and SWLS scales. Logistic regression analysis was used to examine associations, and Baron and Kenny's mediation test and Sobel test were used to assess the mediating effect of life satisfaction.

**Results**

Engaging in agricultural work was positively associated with increased depressive symptoms scores (B = 3.437, p < 0.001), indicating that agricultural work exacerbates depressive symptoms. This effect was partially mediated by life satisfaction.

**Conclusions**

Self-employed agricultural workers face a higher risk of depressive symptoms and are a high-risk group for depressive symptoms. Additionally, life satisfaction plays a mediating role between type of jobs and depressive symptoms. Public health recommendations aimed at improving or mitigating depressive symptoms among agricultural workers could focus on enhancing life satisfaction to promote healthier psychological status.

**Key Words:** Agriculture work; Depressive symptoms; Life satisfaction; CHARLS; China

#### Introduction

Depression is a common mental illness and is now the biggest health burden worldwide[1, 2]. According to the World Health Organization, about 5 % of adults suffer from depression worldwide, amounting to 280 million people, of which more than 54 million people in China suffer from depression[3] . The number of people suffering from depression is still on the rise with each passing year. Given the impact of depression on individuals and society, there is a great need for in-depth research on depression.

Research has shown that work is closely related to the development of depression[4], which can be subdivided into work pressure[5], work hours[6, 7] , work environment[8, 9],work support[10],subjective social status[11]and so on, but there is not that much research focusing on the association between job type—agricultural and non-agricultural—and depression. Among the mainstream treatments for depression, there is a therapy called Horticultural Therapy[12, 13], which suggests that gardening and being close to nature can help people with depression to recover.While empirical research has demonstrated a significantly elevated prevalence of depressive symptoms among middle-aged and elderly populations residing in rural China compared to their urban counterparts[14]. This epidemiological phenomenon stands in stark contrast to the theoretical postulates of Horticultural Therapy, which advocate the mental health benefits derived from nature immersion. The findings reveal a complex mechanistic relationship between agrarian occupational environments and psychological well-being, indicating that mere proximity to natural elements fails to comprehensively elucidate the etiological pathways through which agricultural labor influences depressive states. Life satisfaction is an important indicator of subjective well-being, which can reflect an individual's cognitive evaluation of quality of life. It has been proved that there is an association between life satisfaction and depression in middle-aged and elderly people[15, 16] , which can be used as an important indicator of mental health and quality of life of middle-aged and elderly people. And some other factors of work have been shown to be associated with life satisfaction[17–19] . Therefore, in this study, it is necessary to investigate whether the type of jobs has a correlation with life satisfaction

This study will first examine the association between different types of work and depressive symptoms. Further, it will analyze population subgroups based on work state—self-employed versus employed—in order to provide more targeted public health recommendations. Additionally, life satisfaction will be incorporated into the model as a mediating variable to explore whether job types influence depressive symptoms through the pathway of life satisfaction. It was hypothesized that agricultural work could reduce depressive symptoms by increasing subjects' life satisfaction. The relationship between farming, life satisfaction and depressive symptoms was empirically investigated as a way to provide a theoretical basis for national mental health promotion.

#### Method

**Research design**

The data comes from the 2015 China Health and Aging Tracking Survey ( CHARLS ), a large-scale social survey project jointly initiated and undertaken by the National Development Research Institute and the School of Management at Peking University. The database aims to collect tracking survey data on the health, economic and social conditions of China's middle-aged and elderly population aged 45 and above, providing important data support for the study of China's aging problem.

The survey in the CHARLS database covers a wide range of aspects such as the health states, lifestyle, economic states, social security, family structure and mental health of middle-aged and elderly people. The survey adopts a multi-stage random sampling method to ensure the representatives of the sample. The questionnaire was rigorously designed, and internationally recognized indicators and measurement methods were used to ensure the comparability of the data. All subjects signed an informed consent form and were approved by the Ethics Committee of Peking University ( IRB00001052-11015 ).

**Ethics approval**

All respondents gave informed written consent prior to interview, and ethical approval for the collection of human subject data was obtained, which was updated annually at the Peking University Institutional Review Board (IRB00001052-11015). No additional ethical approval is required for approved data users. All methods of this study were performed in accordance with the guidelines of the Declaration of Helsinki.

**Study population**

In the present study, participants were from CHARLS (2015) and the criteria for inclusion of subjects at baseline were: subjects who contained a record of type of jobs, assessment of depressive states, assessment of life satisfaction, age, gender, type of residence, work states, marital states, socially engaged activity score, smoking states, drinking states, sleep duration, prior work states in CHARLS 2015 . Of these, missing values for job type records (n = 23), missing values for depressive symptoms ( n = 1297) missing values for life satisfaction (n = 1436), and missing values for other covariates (n = 11270) were excluded, and 6856 subjects were ultimately enrolled in the study (Fig. 1).

**Measurement tools**

**Job type and Work state**

In this study, the population was grouped by type of jobs and work states according to the questionnaire questions of CHARLS 2015 F WORK, RETIREMENT AND PENSION. The type of jobs was categorized into those engaged in agricultural work and those engaged in non-agricultural work, and the work states was categorized into self-employed and employed. The exact division steps can be seen in the following figure (Fig. 2).

**Depressive symptoms**

Depression states was measured using the 10-item Center for Epidemiological Studies Depression Scale (CES-D), which is now a widely used and proven valid and reliable common indicator of depression[20, 21]. Previous studies have shown that this assessment is a sensitive, responsive, valid, and reliable tool for identifying and tracking depressive symptoms in Chinese adults.The CES-D assesses depression through 10 dimensions, including 8 negative emotions (e.g., I feel depressed) and 2 positive emotions (e.g., I am hopeful about the future). Participants were asked to assess how often they felt each of the ten emotions in the past week. Responses to each dimension were scored and coded on a 4-point scale ranging from 0 "little or no time ( < 1 day)" to 3 "most or all of the time ( 5 - 7 days)". The final CES - D score was calculated by combining the ten dimensions, ranging from 0 - 30, and was corrected for two positive emotions.The higher the CES - D score, the more severe the depression.

**Life satisfaction**

Life satisfaction was assessed at baseline by asking participants "How satisfied are you with your life in general?". "Life satisfaction was assessed at baseline. This question correlates strongly with the validated 5-item Satisfaction with Life Scale (SWLS)[22] and has been used extensively in previous studies. The five response options are: not at all satisfied, not too satisfied, relatively satisfied, very satisfied, and extremely satisfied. The scores range from 0 ~ 4, with higher scores indicating higher life satisfaction.

**Selection of covariates**

Based on previous studies, we selected a series of covariates including demographic characteristics such as age[23–25], gender[26, 27], type of residential address[28], and marital states[29, 30], as well as factors that have been validated by research to be highly correlated with depressive states, such as social activity participation[31], smoking states[32–34], drinking states[35]. ~~and work hours~~. These covariates were obtained from CHARLS: gender was categorized as 0 (female) and 1 (male), type of residential address was categorized as 0 (urban) and 1 (rural), and marital states was categorized as 0 (unmarried) and 1 (married), and social activity participation was assessed by using the question DA056 in CHARLS 2015, which asked subjects "In the past month, did you engage in the following Social activities" was asked in question DA056 of CHARLS 2015, and scores were assigned according to the social activities selected, with each item accumulating one point, ranging from 0 to 11 points, with higher scores indicating higher participation in social activities. Smoking states was categorized as 0 (non-smokers), 1 (smokers); drinking states was rated using a continuous value score, based on question DA067 in the self-answered CHARLS 2015, with scores ranging from 0 ~ 2, with higher scores indicating more frequent drinking. Prior work states data were derived from the ZF1 loading variable of CHARLS 2015, categorized as 0 (not working at the time of the last round of the survey), 1 (working in non-farming at the time of the last round of the survey), 2 (working in agriculture at the time of the last round of the survey), and 3 (working in both agriculture and non-farming at the time of the last round of the survey).

**Methods of statistical analysis**

As a preliminary analysis, a presentation of baseline data was conducted. Correlation analyses were then conducted to reveal associations between job type, life satisfaction, and depressive states, as well as with covariates. After clarifying the correlations between variables, we dichotomized depression states for logistic regression and divided the population by job type and job states for subgroup regression analysis. We then conducted mediation effects analyses and performed sensitivity tests. We used Baron and Kenny's mediation test[36, 37] to test the mediating role of life satisfaction. Sobel's test[37, 38] was also used to verify the sensitivity of the mediating effect. Finally, we propose a mediated effects model. The above statistical analysis is based on R-4.3.1.

#### Result

**Sample characteristics before Matching**

The socio-demographic characteristics of the study subjects are shown in Table 1. A total of 6856 subjects were included in this study, of which 1663 were engaged in non-agricultural work and 5193 in agricultural work. A significantly higher proportion of subjects were females (48.8%) working in agriculture than those working in non-agriculture (38.7%), and the mean age of agricultural workers (68.2 years) was higher than that of non-agricultural workers (64.3 years). Most of the subjects working in agriculture had rural residential address (89.9%) and self-employed working states (90.5%), while most of the subjects working in non-agriculture had urban residential address (57.3%) and employed working states (68.9%). There was no significant difference in the marital states of the subjects.

The mean CES-D scores of agricultural workers were significantly higher than those of non-agricultural workers (agricultural: 8.37, non-agricultural: 6.17). In addition to this, there were significant differences between the two groups of subjects in life satisfaction score (p=0.0108), social activity participation score (p<0.001), smoking states (p=0.00442), drinking states (p<0.001), sleep duration (p=0.0263), and prior work states (p<0.001).

**Table 1 Baseline before matching**

|  | **Non-agriculture**  **(N=1663)** | **Agriculture**  **(N=5193)** | **p-value** |
| --- | --- | --- | --- |
| **Gender** |  |  |  |
| Female | 643 (38.7%) | 2532 (48.8%) | <0.001 |
| Male  **Age** | 1020 (61.3%) | 2661 (51.2%) |  |
| Mean (SD) | 64.3 (6.85) | 68.2 (8.30) | <0.001 |
| Median [Min, Max]  **Address_type** | 63.0 [28.0, 91.0] | 68.0 [28.0, 101] |  |
| City | 953 (57.3%) | 526 ( 10.1%) | <0.001 |
| Village  **Work_state** | 710 (42.7%) | 4667 (89.9%) |  |
| Self-employed | 518 (31.1%) | 4701 (90.5%) | <0.001 |
| Employed  **Martial_states** | 1145 (68.9%) | 492 (9.5%) |  |
| Unmarried | 119 (7.2%) | 448 (8.6%) | 0.0651 |
| Married  **CES_D_score** | 1544 (92.8%) | 4745 (91.4%) |  |
| Mean (SD) | 6.17 (5.30) | 8.37 (6.38) | <0.001 |
| Median [Min, Max]  **Life_satisfaction_score** | 5.00 [0, 29.0] | 7.00 [0, 30.0] |  |
| Mean (SD) | 2.44 (0.735) | 2.39 (0.774) | 0.0108 |
| Median [Min, Max]  **Social_activity_score** | 2.00 [0, 4.00] | 2.00 [0, 4.00] |  |
| Mean (SD) | 1.66 ( 1.19) | 1.31 (0.795) | <0.001 |
| Median [Min, Max]  **Smoke_state** | 1.00 [ 1.00, 9.00] | 1.00 [0, 8.00] |  |
| Nonsmoker | 1076 (64.7%) | 3557 (68.5%) | 0.00442 |
| Smoker  **Drinking_state** | 587 (35.3%) | 1636 (31.5%) |  |
| Mean (SD) | 0.823 (0.926) | 0.694 (0.907) | <0.001 |
| Median [Min, Max]  **Sleep_time** | 0 [0, 2.00] | 0 [0, 2.00] |  |
| Mean (SD) | 6.50 ( 1.56) | 6.40 ( 1.95) | 0.0263 |
| Median [Min, Max]  **Previous_work_type** | 7.00 [0, 12.0] | 6.50 [0, 15.0] |  |
| Not Work | 219 ( 13.2%) | 438 (8.4%) | <0.001 |
| Non-agricultural Work | 1047 (63.0%) | 261 (5.0%) |  |
| Agricultural Work | 218 ( 13.1%) | 3687 (71.0%) |  |
| Both | 179 ( 10.8%) | 807 ( 15.5%) |  |

**Sample Characteristics after Matching**

After propensity matching scores, the socio-demographic characteristics of the study subjects after matching those who worked in agriculture and those who did not are shown in Table 2. 1,663 people worked in non-agricultural jobs and 1,663 people worked in agriculture after matching. Agricultural workers had a higher proportion of females (69.3% vs. 38.7%, p < 0.001) and a higher mean age (76.1 years vs. 64.3 years, p < 0.001) compared to non-agricultural workers. All agricultural workers lived in rural areas and were mostly self-employed (93.1%), while most non-agricultural workers lived in urban areas (57.3%) and were employed (68.9%). There was no significant difference in the marital states of the subjects. The mean CES-D scores of agricultural workers were significantly higher than those of non-agricultural workers (agricultural: 9.79, non-agricultural: 6.17).

**Table 2 Baseline after matching**

|  | **Non-agriculture**  **(N=1663)** | **Agriculture (N=1663)** | **P-value** |
| --- | --- | --- | --- |
| **Gender** |  |  |  |
| Female | 643 (38.7%) | 1152 (69.3%) | <0.001 |
| Male  **Age** | 1020 (61.3%) | 511 (30.7%) |  |
| Mean (SD) | 64.3 (6.85) | 76.1 (5.98) | <0.001 |
| Median [Min, Max]  **Address_type** | 63.0 [28.0, 91.0] | 76.0 [65.0, 101] |  |
| City | 953 (57.3%) | 0 (0%) | <0.001 |
| Village | 710 (42.7%) | 1663 ( 100%) |  |
| **Work_state** |  |  |  |
| Self-employed | 518 (31.1%) | 1548 (93.1%) | <0.001 |
| Employed | 1145 (68.9%) | 115 (6.9%) |  |
| **Martial_status** |  |  |  |
| Unmarried | 119 (7.2%) | 200 ( 12.0%) | <0.001 |
| Married | 1544 (92.8%) | 1463 (88.0%) |  |
| **CES_D_score** |  |  |  |
| Mean (SD) | 6.17 (5.30) | 9.79 (6.86) | <0.001 |
| Median [Min, Max] | 5.00 [0, 29.0] | 8.00 [0, 30.0] |  |
| **Life_satisfaction_score** |  |  |  |
| Mean (SD) | 2.44 (0.735) | 2.37 (0.770) | 0.00708 |
| Median [Min, Max]  **Social_activity_score** | 2.00 [0, 4.00] | 2.00 [0, 4.00] |  |
| Mean (SD) | 1.66 ( 1.19) | 1.08 (0.458) | <0.001 |
| Median [Min, Max]  **Smoke_state** | 1.00 [ 1.00, 9.00] | 1.00 [0, 5.00] |  |
| Nonsmoker | 1076 (64.7%) | 1324 (79.6%) | <0.001 |
| Smoker | 587 (35.3%) | 339 (20.4%) |  |
| **Drinking_state** |  |  |  |
| Mean (SD) | 0.823 (0.926) | 0.515 (0.840) | <0.001 |
| Median [Min, Max]  **Sleep_time** | 0 [0, 2.00] | 0 [0, 2.00] |  |
| Mean (SD) | 6.50 ( 1.56) | 6.10 (2.20) | <0.001 |
| Median [Min, Max]  **Previous_work_type** | 7.00 [0, 12.0] | 6.00 [0, 15.0] |  |
| Not Work | 219 ( 13.2%) | 206 ( 12.4%) | <0.001 |
| Non-agricultural Work | 1047 (63.0%) | 28 ( 1.7%) |  |
| Agricultural Work | 218 ( 13.1%) | 1343 (80.8%) |  |
| Both | 179 ( 10.8%) | 86 (5.2%) |  |

**Relevance analysis**

In order to understand the relationship between the variables and the mechanism of action, the Spearman's correlation analysis of the variables is shown in Fig. 3. Overall, age was positively correlated with type of job (r=0.68,p<0.05), type of residence (r=0.49 , p<0.05) and social activity participation score (r=0.25 , p<0.05) and with marital states (r=-0.18 , p<0.05), CES-D score (r=-0.22 ,p <0.05) and prior work states (r=-0.49 , p<0.05), while there was no correlation with gender, life satisfaction score, smoking states, drinking states and sleep duration.

**Logistic regression**

Next, in order to explain the relationship between the variables and the depressive state and to find the risk factors for the depressive state, a Logistic multi-factorial regression model was developed (Table 3). Logistic regression analysis revealed that being male (OR = 0.52, 95% CI 0.37 to 0.72, p < 0.001), married (OR = 0.47, 95% CI 0.34 to 0.64, p < 0.001), and employed (OR = 0.56, 95% CI 0.40 to 0.79, p < 0.001) were significantly protective against depressive states. Additionally, life satisfaction (OR = 0.41, 95% CI 0.36 to 0.48, p < 0.001), social activity participation (OR = 0.60, 95% CI 0.47 to 0.76, p < 0.001), and sleep duration (OR = 0.77, 95% CI 0.73 to 0.81, p < 0.001) had significant protective effects against depressive states.

**Table 3 Logistic Regression**

| **Dependent: CES-D score** | | | **Low (N=2858)** | **High (N=468）** | **OR (multivariable)** |
| --- | --- | --- | --- | --- | --- |
| **Job type** | Non-agriculture | | 1554 (54.4%) | 109 (23.3%) |  |
|  | Agriculture | | 1304 (74%) | 359 (76.7%) | 1.07 (0.68-1.68, p=.774) |
| **Gender** | Female | | 1443 (50.5%) | 352 (75.2%) |  |
|  | Male | | 1415 (49.5%) | 116 (24.8%) | 0.52 (0.37-0.72, p<.001) |
| **Age** | Mean ± SD | | 69.7 ± 8.8 | 73.3 ± 7.5 | 1.01 (0.99-1.03, p=.422) |
| **Address type** | City | | 896 (31.4%) | 57 ( 12.2%) |  |
|  | Village | | 1962 (68.6%) | 411 (87.8%) | 1.13 (0.74-1.73, p=.571) |
| **Work state** | Self-employed | | 1678 (58.7%) | 388 (82.9%) |  |
|  | Employed | | 1180 (41.3%) | 80 ( 17.1%) | 0.56 (0.40-0.79, p<.001) |
| **Martial status** | Unmarried | | 226 (7.9%) | 93 ( 19.9%) | 0.47 (0.34-0.64, p<.001) |
|  | Married | | 2632 (92.1%) | 375 (80.1%) |  |
| **Life satisfaction score** | | Mean ± SD | 2.5 ± 0.7 | 1.9 ± 0.9 | 0.41 (0.36-0.48, p<.001) |
| **Social activity score** | Mean ± SD | | 1.4 ± 1.0 | 1.1 ± 0.4 | 0.60 (0.47-0.76, p<.001) |
| **Smoke state** | Nonsmoker | | 2023 (70.8%) | 377 (80.6%) |  |
|  | Smoker | | 835 (29.2%) | 91 ( 19.4%) | 1.24 (0.89-1.72, p=.201) |
| **Drinking state** | Mean ± SD | | 0.7 ± 0.9 | 0.4 ± 0.8 | 0.88 (0.76-1.02, p=.098) |
| **Sleep time** | Mean ± SD | | 6.5 ± 1.8 | 5.2 ± 2.2 | 0.77 (0.73-0.81, p<.001) |
| **Previous work type** | Not Work | | 349 ( 12.2%) | 76 ( 16.2%) |  |
|  | Non-agricultural Work | | 1020 (35.7%) | 55 ( 11.8%) | 0.50 (0.32-0.79, p=.003) |
|  | Agricultural Work | | 1248 (43.7%) | 313 (66.9%) | 0.93 (0.67-1.29, p=.673) |
|  | Both | | 241 (8.4%) | 24 (5.1%) | 0.64 (0.38-1.09, p=.099) |

**Subgroup regression analysis**

In order to explore in more depth the differences in risk factors for depressive states across populations, we divided the population into six subgroups according to two categorization criteria - job type and job states.

**Job type subgroups**

The subjects were divided into two subgroups, agricultural and non-agricultural, by type of jobs and the results of the logistic regression are shown in Fig. 4. As can be seen from the figure, subjects working in non-agricultural jobs compared to those working in agricultural jobs had an approximately 1.80-fold increased risk of developing a depressive state in smokers compared to non-smokers (OR = 1.80 ; 95%CI 1.01 to 3.20; p = 0.047). However men (Agricultural: OR = 0.56 ; 95%CI 0.37 to 0.85; p < 0.01 Non-agricultural: OR = 0.39 ; 95%CI 0.22 to 0.69 ; p < 0.001), married (Agricultural: OR = 0.54 ; 95%CI 0.37 to 0.69; p < 0.001), and non-smokers were at greater risk than non-smokers. 95%CI 0.37 to 0.77; p < 0.001 Non-agricultural: OR = 0.33 ; 95%CI 0.18 to 0.60 ; p < 0.001), employed (Agricultural: OR = 0.39 ; 95%CI 0.20 to 0.74; p < 0.01), and married (Agricultural: OR = 0.39 ; 95%CI 0.20 to 0.74; p < 0.01). Non-agricultural: OR = 0.64 ; 95%CI 0.41 to 0.99 ; p < 0.05), life satisfaction (Agricultural: OR = 0.47 ; 95%CI 0.39 to 0.56 ; p < 0.001 Non-agricultural: OR = 0.30 ; 95%CI 0.22 to 0.56 ; p < 0.001 95%CI 0.22 to 0.42 ; p < 0.001), participation in social activities (Agricultural: OR = 0.61 ; 95%CI, 0.42-0.88; p < 0.01 Non-agricultural: OR = 0.52; 95%CI 0.36 to 0.76 ; p < 0.001), and sleep duration. 0.001) and sleep duration (Agricultural: OR = 0.78 ; 95%CI 0.74 to 0.83; p < 0.001 Non-agricultural: OR = 0.71; 95%CI 0.62 to 0.82; p < 0.001) were significant protective factors for both subgroups.

**Work state subgroups**

Subjects were categorized into four subgroups, self-employed non-farmers (A), self-employed farmers (B), employed non-farmers (C), and employed farmers (D), based on the dual criteria of job states and job type, and the results of the logistic regression are shown in Fig. 5.

As can be seen from the figure, in the group with non-agricultural jobs, self-employed subjects had only two significant protective factors for life satisfaction (OR = 0.24 ; 95%CI 0.14 to 0.42 ; p < 0.001) and sleep duration (OR = 0.75 ; 95%CI 0.60 to 0.93; p = 0.01) in comparison to employed subjects, while employed workers had only two significant protective factors in addition to life satisfaction (OR = 0.35 ; 95%CI 0.24 to 0.51; p < 0.001) and sleep duration (OR = 0.69 ; 95%CI 0.58 to 0.82; p < 0.001), there were two significant protective factors for employed workers in addition to male (OR = 0.28 ; 95%CI 0.12 to 0.63; p < 0.01), married (OR = 0.26 ; 95%CI 0.13 to 0.53; p < 0.001) and social activity participation (OR = 0.45 ; 95%CI 0.27 to 0.76; p < 0.01), which were three specific protective factors.

In contrast, in the group working in agriculture, there were no significant protective factors for employed subjects, while men (OR = 0.53 ; 95%CI 0.35 to 0.81; p < 0.01), married (OR = 0.52 ; 95%CI 0.36 to 0.76 ; p < 0.001), life satisfaction (OR = 0.45 ; 95%CI 0.38 to 0.54 ; p < 0.001), social activity participation (OR = 0.64 ; 95%CI 0.43 to 0.95; p < 0.05), and sleep duration (OR = 0.78 ; 95%CI 0.73 to 0.82; p < 0.001) were significant protective factors for self-employed workers.

**Mediation model analysis and test results**

Baron and Kenny's stepwise regression was used to verify the mediating role of life satisfaction between job type (agricultural vs. non-agricultural) and depressive states. The total effect of job type on depressive states (Path c) was significant (B = 3.622, p < 0.001). Life satisfaction scores showed a significant negative correlation with job type (Path a: B = -0.070, p < 0.001) and depressive states (Path b: B = -2.641, p < 0.001). The indirect effect mediated by life satisfaction (Path a*b) was 0.186 (SE = 0.070, 95% CI 0.0492 to 0.320, p < 0.01), indicating a significant indirect effect. After controlling for the mediator variable life satisfaction, the coefficient of job type on depressive states (Path c': B = 3.437, p < 0.001) remained significant, indicating that the direct effect was also significant and that the effect of job type on depressive states was partially mediated by life satisfaction.The final mediation model is shown in Fig. 6.

As shown in Table 5, the estimate of ACME (Average Causal Mediation Effects) was 0.1858, indicating that life satisfaction as a mediating variable produced a significant positive indirect effect between job type and depressive state, suggesting that agricultural workers increased their depressive state by 0.1858 standard deviations through life satisfaction. The mediating effect explained 5.13% of the total effect (B = 3.6224 , p < 0.001), suggesting that the level of life satisfaction scores played a role in explaining the role of job type on depressive states.

**Table 4 Mediation Analysis**

| **Variable** | **Path c** | | **Path a** | | **Path c’ and Path b** | | **Path a*b** | | | |
| --- | --- | --- | --- | --- | --- | --- | --- | --- | --- | --- |
|  | **B** | **SE** | **B** | **SE** | **B** | **SE** | **B** | **SE** | **LLCI** | **ULCI** |
|  | 3.622*** | 0.213 | -0.070*** | 0.026 | 3.437*** | 0.201 | 0.186** | 0.070 | 0.0492 | 0.320 |

Note:*p < 0.05;**p < 0.01; ***p < 0.001

**Table 5 Causal Mediation Analysis**

|  | **Estimate** | **95% CI Lower** | **95% CI Upper** | **p-value** |
| --- | --- | --- | --- | --- |
| **ACME** | 0.1858 | 0.0492 | 0.32 | 0.002** |
| **ADE** | 3.4366 | 3.0224 | 3.84 | < 0.001*** |
| **Total Effect** | 3.6224 | 3.2044 | 4.03 | < 0.001*** |
| **Prop.Mediated** | 0.0513 | 0.0141 | 0.09 | 0.002** |

Note:*p < 0.05; **p < 0.001

The sensitivity analysis results are shown in Figure Fig. 7. The X-axis is the sensitivity parameter Rho and the Y-axis is the value of the mediating effect. When the point estimate of the average causal mediating effect ACME is 0, i.e., the absolute value of rho when the mediating effect disappears is 0.3, which indicates that the confounding effect is stronger, i.e., the results of this mediating effect model are more reliable.

#### Discussion

This cross-sectional study looks at the effect of job type - i.e., whether or not one works in agriculture - on depressive states among middle-aged and older adults over the age of 45. Based on a series of regression and mediation analyses from the CHARLS national survey, the findings suggest that agricultural workers have significantly higher depression scores than non-agricultural workers, while life satisfaction partially mediates the association between job type and depression.

Our findings indicate that subjects working in agriculture had higher mean CES-D scores than those working in non-agricultural jobs, suggesting that depressive states are more severe in agricultural jobs, which is not consistent with our hypothesis based on horticultural therapy. Through further Logistic multifactorial regression analysis, we could observe that the type of jobs did not play a role in the occurrence of depressive states under the consideration of the inclusion of other covariates, suggesting that the facilitating effect of agricultural work on depressive states was in the presence of other exposure factors. Previous studies have indicated that exposure factors such as pesticide exposure[39–41], social activities [42], and social support [43] are associated with depressive states among agricultural workers, whereas in the present study, the researcher's baseline showed that life satisfaction scores and social activity participation scores of agricultural workers were lower than those of non-agricultural workers, and further subgroup regression analyses of the type of job with or without farming it can be observed that the increase in life satisfaction and social activity participation of agricultural workers reduces the risk of the occurrence of depressive states. Thus, we can propose the idea that lower life satisfaction and social activity participation are risk factors for the development of depression among agricultural workers.The reasons for low life satisfaction among agricultural workers are complex, primarily influenced by the following factors:(1) Economic Pressure[41, 44]: Agricultural workers’ incomes are generally less stable compared to those in non-agricultural occupations, as they are highly susceptible to weather changes, market fluctuations, and other external factors. The seasonal and uncertain nature of agricultural income can impose substantial financial stress, leading to lower life satisfaction.(2) Occupational Health Risks[45]: Agricultural workers face high occupational health risks, including long working hours, exposure to adverse weather conditions, and pesticide exposure mentioned earlier. These health risks negatively impact both physical and mental well-being, reducing overall life satisfaction.(3) Social Isolation and Lack of Social Support[44]: Agricultural workers are often geographically isolated due to the nature of their work, resulting in limited social interactions and relationships. This isolation contributes to lower levels of social participation. The lack of social support and limited opportunities for interaction may lead to feelings of loneliness and depressive symptoms, further impacting life satisfaction.(4) Mental Health Issues and Cultural Stigma[45]: In rural areas, mental health issues are still subject to cultural stigma, which makes it difficult for agricultural workers experiencing mental health challenges to seek help. The lack of mental health support resources can exacerbate these issues, leading to further declines in life satisfaction.

The baseline shows that compared to non-agricultural workers, agricultural workers live in rural areas. Urbanization has been a trend in the world, and China has followed the world trend and entered the process of rapid urbanization [46–48], which has led to the center of gravity of socio-economic activities being located in the city, resulting in a dramatic change in living environments and living factors. Therefore, the difference between urban and rural life affects mental health to a certain extent, and according to existing studies [49, 50] we can point out that the main reason for this effect is that the difference between urban and rural living space and socio-economics makes urban areas have more diversified social activities and more abundant social resources compared to rural areas, which leads to a significant increase in the life satisfaction of urban residents. The social resources available in cities include education[49], health care services [51], pension benefits[49], social infrastructure, and so on. A high level of education can make middle-aged and elderly people have good adaptability in the face of adversity, and has been shown that the education of middle-aged and elderly people will affect the education of their own offspring, which in turn affects the intergenerational support for the elderly, and thus has an impact on the symptoms of depression among the elderly[52]; The lack of medical resources in rural areas prevents middle-aged and elderly people from seeking timely medical treatment when they face a series of health problems, which can lead to functional disabilities, and it has been pointed out in an empirical study that functional disabilities of middle-aged and elderly people reduce life satisfaction and aggravate their depressive symptoms [16]; health insurance and pension insurance coverage is higher in urban areas, which can alleviate the economic pressure to a certain extent. This suggests that living in cities helps to increase life satisfaction[53]. There is also evidence from Asia that participation in social activities to increase social engagement has a positive impact on the mental health of middle-aged and older adults [42, 51] .Current studies indicate a significant negative correlation between digital village construction and depressive symptoms among rural middle-aged and older adults, with digital village construction alleviating depression through income, consumption, and cognitive welfare effects[14] . Based on these findings, the following measures can effectively leverage the three welfare effects of digital village construction to reduce depressive risks among rural agricultural workers and promote healthy aging: 1) enhancing digital infrastructure (e.g., improving rural internet coverage and e-commerce platforms to boost income)[54]; 2) organizing digital literacy training to strengthen cognitive abilities[55, 56]; 3) optimizing logistics and online services to facilitate consumption[57].

Our findings suggest that males are a protective factor for the occurrence of depressive states in both the farming and non-farming populations, indicating that there is a gender difference in the occurrence of depressive states and that females are at a higher risk for their occurrence, which is consistent with the findings of previous studies[58–60]. There are many factors that contribute to the high prevalence of depressive states in the female population, including a combination of biological, psychological, and sociocultural factors. Biologically, women are more sensitive to hormonal fluctuations associated with the reproductive cycle, such as hormonal fluctuations (menstrual cycle, postpartum, and menopause) that can affect mood thus leading to the occurrence of premenstrual irritability disorder and postpartum depressive states, among others[59–61]; psychology points out that women are more prone to recurring thoughts of negativity[59], which may be associated with an increased risk of depressive states; and in the process of growing up, the Gender roles and societal expectations place additional stress on women, with sexual violence [62], sexual abuse [60], interpersonal violence, stress exposure and stress susceptibility [33] at the micro level, and gender inequality[63, 64] at the macro level, all of which increase the risk for the development of depressive states in women. Marital states can also have a significant impact on an individual's emotional health, and there have been precedent results demonstrating that marriage is a protective factor for depressive states[65–67], a finding that is consistent with our findings. There is a positive correlation between marriage and mental health, the main reason is that marriage provides emotional fulfillment and social support, the intimate relationship between marriage partners can provide each other with emotional support and encouragement, help each other to cope with setbacks and difficulties in life, reduce loneliness and social isolation, and enhance mental health. In addition to this marriage implies the sharing of economic resources such as housing, income and property, which can reduce financial stress and anxiety. However, it is worth noting that marital quality plays an important role in moderating the effects of marriage on mental health [65].

In order to better provide targeted recommendations for the adjustment of workers' depressive state, we further conducted subgroup regression analysis based on whether or not they were self-employed, and the results showed that in the agricultural population, self-employed people had more protective factors than employed people, while in the non-agricultural population, employed people had more protective factors than self-employed people. According to the conclusion of the previous study [58], self-employed workers have a greater risk of depression, we analyze that the causes of depression among self-employed workers may lie in the following points: 1 ) Unstable source of economic income: self-employed workers, whether they work in agriculture or non-agriculture, need to be responsible for their own work profit and loss, and the economic pressure is high, while in comparison with the employed workers' source of economic income is more stable, with better welfare benefits such as health insurance and health care, and the benefits are better. better welfare benefits, such as higher coverage of health insurance and pension insurance[68]; 2) blurred work-life boundaries: the uncertainty of working hours often takes up time for private life, resulting in insufficient time for rest and relaxation; 3) high work intensity: in order to maintain continuity and development of their work business, self-employed workers need to spend more time and energy than employed workers; 4) self-employment is mostly in agriculture, construction, mining, etc., and poorer workplace exposure exposes workers to higher physical and mental health risks[58, 69]; 5) and self-employed workers usually lack social support from organizations, which also increases their risk of depression[58]. For the agricultural self-employed and non-agricultural employed people, there are several suggestions we proposed: 1) Improve life satisfaction by improving the quality of life[70, 71]; 2) Participate in more social activities to enhance the interaction with friends and social support[42, 43, 51]; 3) Extend the sleep time and develop good sleep habits to get sufficient physical and mental rest and relaxation[72–74]. Moreover, for the government to complement public health policies for agricultural workers, we proposed following suggestions: 1) Construct more rural fitness and recreational facilities; 2) Conduct more mental health screening activities in rural areas; 3) Implement policies to improve the incomes of agricultural workers in rural areas.

According to the results of BK stepwise regression, it can be observed that there is a partial mediating effect of life satisfaction between the level of depressive state and the type of jobs, i.e., the negative correlation between life satisfaction and the type of jobs indicates that the life satisfaction of agricultural workers is lower than that of non-agricultural workers, and the negative correlation between life satisfaction and depressive state indicates that the risk of depressive state can be reduced by improving life satisfaction, which indicates that agricultural workers have low life satisfaction due to low depressive state. The negative correlation between life satisfaction and depression indicates that the risk of depression can be reduced by increasing life satisfaction, suggesting that agricultural workers are depressed due to low life satisfaction. In terms of the mechanism of depression, studies have shown that there is a close association between depression and life satisfaction, and that the occurrence of depressive symptoms is often accompanied by a decrease in life satisfaction[75], and people with low life satisfaction are more likely to experience depression[76]; the results of a priori RCT have shown that depression symptoms can be significantly reduced by increasing life satisfaction[50], and the results of another RCT found that an active psychological intervention, if conducted for a year, was effective in reducing depressive symptoms and increasing life satisfaction[75]. All of these findings further support the results of our mediation analysis and are consistent with the findings of the regression analysis.

Longitudinal designs are essential for understanding the temporal relationship between job type and mental health outcomes. Existing longitudinal investigations have illuminated that the depressive symptoms is not static; rather, it can fluctuate over time, which cross-sectional studies may not adequately represent. Longitudinal research highlights the dynamic relationship between job type and mental health, with studies showing that unstable employment can lead to increased depressive symptoms over time[77] and work-related stressors can exacerbate mental health issues[78]. These findings underscore the necessity of longitudinal studies to accurately capture these evolving dynamics.

#### Conclusion

Our study investigated the relationship between the type of jobs, whether farming or not, and depressive states, emphasizing through subgroup analysis that self-employed job type, agricultural job and sex as female are risk factors of developing depressive states and developing depression. In addition, life satisfaction mediated the relationship between the type of jobs and the level of depressive state, a result that highlights the complex association between the type of jobs and depressive state.

Therefore, based on the results of our logistic regression and mediation analyses, public health recommendations that can intervene in the occurrence of depressive states or alleviate existing depressive states by improving life satisfaction can be made for agricultural workers in order to help agricultural workers have a healthier psychological state. Future research could focus on the mechanisms by which differences in specific parameters of life satisfaction under different job type classifications affect depressive states and whether other mediating variables are also present.

Finally, the study has limitations, including using cross-sectional data to examine the relationship between job type and depressive states, relying on a self-reported depression scale, and not being sufficiently popularized in China for mental disorder treatment. Additionally, the number of covariates included was too large to fully reveal the effects of each factor on depressive states under job type classification. Further research should use longitudinal data to reveal causal effects.

#### Declarations

**Funding**

This research is supported by Key Laboratory of Philosophy and Social Sciences of Colleges and Universities in Guangdong Province (2023WSYS005, 2023WSYS007), the 13th Five-Year Plan of Guangdong Province for Philosophy and Social Sciences (GD20XGL42).

**Competing Interests**

The authors declare that they have no known competing financial interests or personal relationships that could have appeared to influence the work reported in this paper.

**Author Contributions**

Feiying He and Yuan Yao Chen contributed to the study conception and design. Data collection and analysis were performed by Xiaoying Li and Xiangchun Xu. The first draft of the manuscript was written by Xiaoying Li and Xiangchun Xu and all authors commented on previous versions of the manuscript. All authors read and approved the final manuscript.

**Consent to publish**

Informed consent was obtained from all individual participants included in the study.

#### Reference

1.	Doris A, Ebmeier K, Shajahan P (1999) Depressive illness. Lancet 354:1369–1375. https://doi.org/10.1016/S0140-6736(99)03121-9

2.	Mental health: A world of depression | Nature. https://www.nature.com/articles/515180a. Accessed 25 Jun 2024

3.	Wu P, Wang R, Lin H, et al (2023) Automatic depression recognition by intelligent speech signal processing: A systematic survey. CAAI Transactions on Intelligence Technology 8:701–711. https://doi.org/10.1049/cit2.12113

4.	Liu Y, Yang X, Wu Y, et al (2023) The Relationship between Job Satisfaction and Depressive Symptoms among Chinese Adults Aged 35-60 Years: The Mediating Role of Subjective Well-Being and Life Satisfaction. Int J Environ Res Public Health 20:2023. https://doi.org/10.3390/ijerph20032023

5.	He S-C, Wu S, Wang C, et al (2018) Interaction between job stress and the BDNF Val66Met polymorphism affects depressive symptoms in Chinese healthcare workers. Journal of Affective Disorders 236:157–163. https://doi.org/10.1016/j.jad.2018.04.089

6.	Song X, Fu W, Liu X, et al (2020) Mental health status of medical staff in emergency departments during the Coronavirus disease 2019 epidemic in China. Brain Behav Immun 88:60–65. https://doi.org/10.1016/j.bbi.2020.06.002

7.	Bryant-Genevier J, Rao CY, Lopes-Cardozo B, et al (2021) Symptoms of Depression, Anxiety, Post-Traumatic Stress Disorder, and Suicidal Ideation Among State, Tribal, Local, and Territorial Public Health Workers During the COVID-19 Pandemic - United States, March-April 2021. MMWR Morb Mortal Wkly Rep 70:947–952. https://doi.org/10.15585/mmwr.mm7026e1

8.	Siegrist J, Wege N (2020) Adverse Psychosocial Work Environments and Depression-A Narrative Review of Selected Theoretical Models. Front Psychiatry 11:66. https://doi.org/10.3389/fpsyt.2020.00066

9.	Shiga K, Izumi K, Minato K, et al (2022) Association of work environment with stress and depression among Japanese workers. Work 72:1321–1335. https://doi.org/10.3233/WOR-210356

10.	Jia CX, Cheung C-K, Fu C (2020) Work Support, Role Stress, and Life Satisfaction among Chinese Social Workers: The Mediation Role of Work-Family Conflict. Int J Environ Res Public Health 17:8881. https://doi.org/10.3390/ijerph17238881

11.	(2024) The relationship between job satisfaction and depressive symptoms in Chinese men: a moderated multiple mediation model. JOMH 20:80. https://doi.org/10.22514/jomh.2024.056

12.	Xu M, Lu S, Liu J, Xu F (2023) Effectiveness of horticultural therapy in aged people with depression: A systematic review and meta-analysis. Front Public Health 11:1142456. https://doi.org/10.3389/fpubh.2023.1142456

13.	Soga M, Gaston KJ, Yamaura Y (2017) Gardening is beneficial for health: A meta-analysis. Prev Med Rep 5:92–99. https://doi.org/10.1016/j.pmedr.2016.11.007

14.	Wan S, Zhang P, Chen S, Yang Y (2025) Digital village construction and depressive symptoms in rural middle-aged and older adults: Evidence from China. J Affect Disord 372:200–209. https://doi.org/10.1016/j.jad.2024.12.026

15.	Gigantesco A, Fagnani C, Toccaceli V, et al (2019) The Relationship Between Satisfaction With Life and Depression Symptoms by Gender. Front Psychiatry 10:419. https://doi.org/10.3389/fpsyt.2019.00419

16.	Li A, Wang D, Lin S, et al (2021) Depression and Life Satisfaction Among Middle-Aged and Older Adults: Mediation Effect of Functional Disability. Front Psychol 12:755220. https://doi.org/10.3389/fpsyg.2021.755220

17.	Ilies R, Schwind KM, Wagner DT, et al (2007) When can employees have a family life? The effects of daily workload and affect on work-family conflict and social behaviors at home. J Appl Psychol 92:1368–1379. https://doi.org/10.1037/0021-9010.92.5.1368

18.	Yang S, Chen L, Bi X (2023) Overtime work, job autonomy, and employees’ subjective well-being: Evidence from China. Front Public Health 11:1077177. https://doi.org/10.3389/fpubh.2023.1077177

19.	Parida S, Aamir A, Alom J, et al (2021) British doctors’ work-life balance and home-life satisfaction: a cross-sectional study. Postgrad Med J postgradmedj-2021-141338. https://doi.org/10.1136/postmj/postgradmedj-2021-141338

20.	Boey KW (1999) Cross-validation of a short form of the CES-D in Chinese elderly. Int J Geriatr Psychiatry 14:608–617. https://doi.org/10.1002/(sici)1099-1166(199908)14:8<608::aid-gps991>3.0.co;2-z

21.	Irwin M, Artin KH, Oxman MN (1999) Screening for depression in the older adult: criterion validity of the 10-item Center for Epidemiological Studies Depression Scale (CES-D). Arch Intern Med 159:1701–1704. https://doi.org/10.1001/archinte.159.15.1701

22.	Cheung F, Lucas RE (2014) Assessing the validity of single-item life satisfaction measures: results from three large samples. Qual Life Res 23:2809–2818. https://doi.org/10.1007/s11136-014-0726-4

23.	Coryell W, Solomon D, Leon A, et al (2009) Does major depressive disorder change with age? Psychol Med 39:1689–1695. https://doi.org/10.1017/S0033291709005364

24.	Hegeman JM, Kok RM, van der Mast RC, Giltay EJ (2012) Phenomenology of depression in older compared with younger adults: meta-analysis. Br J Psychiatry 200:275–281. https://doi.org/10.1192/bjp.bp.111.095950

25.	Zhu Y, Li C, Xie W, et al (2022) Trajectories of depressive symptoms and subsequent cognitive decline in older adults: a pooled analysis of two longitudinal cohorts. Age Ageing 51:afab191. https://doi.org/10.1093/ageing/afab191

26.	Piccinelli M, Wilkinson G (2000) Gender differences in depression: Critical review. The British Journal of Psychiatry 177:486–492. https://doi.org/10.1192/bjp.177.6.486

27.	Parker G, Brotchie H (2010) Gender differences in depression. Int Rev Psychiatry 22:429–436. https://doi.org/10.3109/09540261.2010.492391

28.	Julien D, Richard L, Gauvin L, Kestens Y (2012) Neighborhood characteristics and depressive mood among older adults: an integrative review. Int Psychogeriatr 24:1207–1225. https://doi.org/10.1017/S1041610211002894

29.	Buckman JEJ, Saunders R, Stott J, et al (2021) Role of age, gender and marital status in prognosis for adults with depression: An individual patient data meta-analysis. Epidemiol Psychiatr Sci 30:e42. https://doi.org/10.1017/S2045796021000342

30.	Min J, Yorgason JB, Fast J, Chudyk A (2020) The Impact of Spouse’s Illness on Depressive Symptoms: The Roles of Spousal Caregiving and Marital Satisfaction. J Gerontol B Psychol Sci Soc Sci 75:1548–1557. https://doi.org/10.1093/geronb/gbz017

31.	Ryu E, Jenkins GD, Wang Y, et al (2023) The importance of social activity to risk of major depression in older adults. Psychol Med 53:2634–2642. https://doi.org/10.1017/S0033291721004566

32.	Pasco JA, Williams LJ, Jacka FN, et al (2008) Tobacco smoking as a risk factor for major depressive disorder: population-based study. Br J Psychiatry 193:322–326. https://doi.org/10.1192/bjp.bp.107.046706

33.	Fluharty M, Taylor AE, Grabski M, Munafò MR (2017) The Association of Cigarette Smoking With Depression and Anxiety: A Systematic Review. Nicotine Tob Res 19:3–13. https://doi.org/10.1093/ntr/ntw140

34.	Munafò MR, Araya R (2010) Cigarette smoking and depression: a question of causation. Br J Psychiatry 196:425–426. https://doi.org/10.1192/bjp.bp.109.074880

35.	Haynes JC, Farrell M, Singleton N, et al (2005) Alcohol consumption as a risk factor for anxiety and depression: Results from the longitudinal follow-up of the National Psychiatric Morbidity Survey. Br J Psychiatry 187:544–551. https://doi.org/10.1192/bjp.187.6.544

36.	Baron RM, Kenny DA (1986) The moderator-mediator variable distinction in social psychological research: conceptual, strategic, and statistical considerations. J Pers Soc Psychol 51:1173–1182. https://doi.org/10.1037//0022-3514.51.6.1173

37.	Wang J, Zhang J, Lin H, et al (2023) Economic development, weak ties, and depression: Evidence from China. Journal of Affective Disorders 334:246–257. https://doi.org/10.1016/j.jad.2023.04.097

38.	Sobel ME (1982) Asymptotic Confidence Intervals for Indirect Effects in Structural Equation Models. Sociological Methodology 13:290. https://doi.org/10.2307/270723

39.	Cancino J, Soto K, Tapia J, et al (2023) Occupational exposure to pesticides and symptoms of depression in agricultural workers. A systematic review. Environ Res 231:116190. https://doi.org/10.1016/j.envres.2023.116190

40.	Frengidou E, Galanis P, Malesios C (2024) Pesticide Exposure or Pesticide Poisoning and the Risk of Depression in Agricultural Populations: A Systematic Review and Meta-Analysis. J Agromedicine 29:91–105. https://doi.org/10.1080/1059924X.2023.2278801

41.	Zanchi MM, Marins K, Zamoner A (2023) Could pesticide exposure be implicated in the high incidence rates of depression, anxiety and suicide in farmers? A systematic review. Environ Pollut 331:121888. https://doi.org/10.1016/j.envpol.2023.121888

42.	Min J, Ailshire J, Crimmins EM (2016) Social engagement and depressive symptoms: do baseline depression status and type of social activities make a difference? Age Ageing 45:838–843. https://doi.org/10.1093/ageing/afw125

43.	Wan Mohd Azam WMY, Din NC, Ahmad M, et al (2013) Loneliness and depression among the elderly in an agricultural settlement: mediating effects of social support. Asia Pac Psychiatry 5 Suppl 1:134–139. https://doi.org/10.1111/appy.12061

44.	Daghagh Yazd S, Wheeler SA, Zuo A (2019) Key Risk Factors Affecting Farmers’ Mental Health: A Systematic Review. Int J Environ Res Public Health 16:4849. https://doi.org/10.3390/ijerph16234849

45.	Research trends in farmers’ mental health: A scoping review of mental health outcomes and interventions among farming populations worldwide | PLOS ONE. https://journals.plos.org/plosone/article?id=10.1371/journal.pone.0225661. Accessed 6 Nov 2024

46.	Bettencourt L, West G (2010) A unified theory of urban living. Nature 467:912–913. https://doi.org/10.1038/467912a

47.	Bai X, Shi P, Liu Y (2014) Society: Realizing China’s urban dream. Nature 509:158–160. https://doi.org/10.1038/509158a

48.	Yang XJ (2013) China’s rapid urbanization. Science 342:310. https://doi.org/10.1126/science.342.6156.310-a

49.	Li LW, Liu J, Xu H, Zhang Z (2016) Understanding Rural-Urban Differences in Depressive Symptoms Among Older Adults in China. J Aging Health 28:341–362. https://doi.org/10.1177/0898264315591003

50.	Stier AJ, Schertz KE, Rim NW, et al (2021) Evidence and theory for lower rates of depression in larger US urban areas. Proc Natl Acad Sci U S A 118:e2022472118. https://doi.org/10.1073/pnas.2022472118

51.	Chang KS (1991) Economic development with limited supplies of family labor: Chinese peasant families in balancing demographic and economic requisites. Korea J Popul Dev 20:47–76

52.	Pei Y, Cong Z, Wu B (2020) Education, adult children’s education, and depressive symptoms among older adults in rural China. Soc Sci Med 253:112966. https://doi.org/10.1016/j.socscimed.2020.112966

53.	Chen L, Chang L, Lin H, et al (2024) Depressive disorder benefits of cities: Evidence from the China. J Affect Disord 350:420–427. https://doi.org/10.1016/j.jad.2024.01.105

54.	Mitchell LM, Joshi U, Patel V, et al (2021) Economic Evaluations of Internet-Based Psychological Interventions for Anxiety Disorders and Depression: A Systematic Review. J Affect Disord 284:157–182. https://doi.org/10.1016/j.jad.2021.01.092

55.	Biagianti B, Foti G, Di Liberto A, et al (2023) CBT-informed psychological interventions for adult patients with anxiety and depression symptoms: A narrative review of digital treatment options. J Affect Disord 325:682–694. https://doi.org/10.1016/j.jad.2023.01.057

56.	Chai Y, Xian G, Wang M, et al (2024) Aging wisely: The impact of Internet use on older adults’ mental health. J Affect Disord 364:139–145. https://doi.org/10.1016/j.jad.2024.08.076

57.	Ji T, Li X, Meng G, et al (2020) The association between banana consumption and the depressive symptoms in Chinese general adult population: A cross-sectional study. J Affect Disord 264:1–6. https://doi.org/10.1016/j.jad.2019.12.008

58.	Chen S, Wang Y, She R (2023) Prevalence and gender disparity of those who screen positive for depression in China by the classification of the employer and industry: a cross-sectional, population-based study. BMC Psychiatry 23:62. https://doi.org/10.1186/s12888-023-04557-7

59.	Kuehner C (2017) Why is depression more common among women than among men? The Lancet Psychiatry 4:146–158. https://doi.org/10.1016/S2215-0366(16)30263-2

60.	Why is depression more prevalent in women? https://psycnet.apa.org/record/2015-41955-001. Accessed 17 Jun 2024

61.	(2013) Diagnostic and statistical manual of mental disorders: DSM-5^TM^, 5th ed. American Psychiatric Publishing, Inc., Arlington, VA, US

62.	Sardinha L, Maheu-Giroux M, Stöckl H, et al (2022) Global, regional, and national prevalence estimates of physical or sexual, or both, intimate partner violence against women in 2018. Lancet 399:803–813. https://doi.org/10.1016/S0140-6736(21)02664-7

63.	Chen Y-Y, Subramanian SV, Acevedo-Garcia D, Kawachi I (2005) Women’s status and depressive symptoms: a multilevel analysis. Soc Sci Med 60:49–60. https://doi.org/10.1016/j.socscimed.2004.04.030

64.	Van De Velde S, Huijts T, Bracke P, Bambra C (2013) Macro‐level gender equality and depression in men and women in Europe. Sociology Health &amp; Illness 35:682–698. https://doi.org/10.1111/j.1467-9566.2012.01521.x

65.	Frech A, Williams K (2007) Depression and the Psychological Benefits of Entering Marriage. J Health Soc Behav 48:149–163. https://doi.org/10.1177/002214650704800204

66.	Umberson D, Montez JK (2010) Social relationships and health: a flashpoint for health policy. J Health Soc Behav 51 Suppl:S54-66. https://doi.org/10.1177/0022146510383501

67.	(2007) The Effects of Marriage on Health: A Synthesis of Recent Research Evidence. Research Brief. In: ASPE. https://aspe.hhs.gov/reports/effects-marriage-health-synthesis-recent-research-evidence-research-brief. Accessed 18 Jun 2024

68.	Huang X (2013) The Politics of Social Welfare Reform in Urban China: Social Welfare Preferences and Reform Policies. J OF CHIN POLIT SCI 18:61–85. https://doi.org/10.1007/s11366-012-9227-x

69.	Wang X, Wu S, Song Q, et al (2011) Occupational health and safety challenges in China--focusing on township-village enterprises. Arch Environ Occup Health 66:3–11. https://doi.org/10.1080/19338244.2010.486424

70.	Mei S, Qin Z, Yang Y, et al (2021) Influence of Life Satisfaction on Quality of Life: Mediating Roles of Depression and Anxiety Among Cardiovascular Disease Patients. Clin Nurs Res 30:215–224. https://doi.org/10.1177/1054773820947984

71.	Chen Y, Hicks A, While AE (2014) Quality of life and related factors: a questionnaire survey of older people living alone in Mainland China. Qual Life Res 23:1593–1602. https://doi.org/10.1007/s11136-013-0587-2

72.	Zhong W, Wang F, Chi L, et al (2022) Association between Sleep Duration and Depression among the Elderly Population in China. Exp Aging Res 48:387–399. https://doi.org/10.1080/0361073X.2021.2008755

73.	Ouyang P, Sun W (2019) Depression and sleep duration: findings from middle-aged and elderly people in China. Public Health 166:148–154. https://doi.org/10.1016/j.puhe.2018.10.007

74.	Sun Y, Shi L, Bao Y, et al (2018) The bidirectional relationship between sleep duration and depression in community-dwelling middle-aged and elderly individuals: evidence from a longitudinal study. Sleep Med 52:221–229. https://doi.org/10.1016/j.sleep.2018.03.011

75.	Lyubomirsky S, King L, Diener E (2005) The benefits of frequent positive affect: does happiness lead to success? Psychol Bull 131:803–855. https://doi.org/10.1037/0033-2909.131.6.803

76.	Miao H, Sun H, He X, et al (2021) Perceived social support and life satisfaction among young Chinese adolescents: the mediating effect of psychological Suzhi and its components. Curr Psychol 40:6164–6174. https://doi.org/10.1007/s12144-020-01130-3

77.	Demiral Y, Ihle T, Rose U, et al (2022) Precarious Work as Risk Factor for 5-Year Increase in Depressive Symptoms. Int J Environ Res Public Health 19:3175. https://doi.org/10.3390/ijerph19063175

78.	Oddo VM, Zhuang CC, Andrea SB, et al (2021) Changes in precarious employment in the United States: A longitudinal analysis. Scand J Work Environ Health 47:171–180. https://doi.org/10.5271/sjweh.3939
