# Supplementary material for: Can working in agriculture have a favorable effect on depressive symptoms? Life satisfaction as a mediator
Source: PLoS One. 2025 Aug 13;20(8):e0325912. doi: 10.1371/journal.pone.0325912 (PMC12349095; doi:10.1371/journal.pone.0325912)
Supplement: Attachment — Submitted filename: renamed_36f54.docx [file pone.0325912.s004.docx]

Dear Editor,

Thank you for your kind decision to have a revision chance! We have thoroughly read any comments made by Reviewers and indeed replied. Thank you again!

Best regards,

Yuan Yao Chen

Reviewer #3:

Dear Reviewer3,

It is our honor to have your approval of our revised manuscript. We really appreciate your precious comments and advice. Thank you again for your highly speak of our work.

• The background is relatively up-to-date, mentioning key studies but could benefit from a broader range of literature around depression and agricultural work.
• The objectives are clearly stated, aiming to explore the association of agricultural work with depressive symptoms and the mediating role of life satisfaction.
• The study methods are sufficiently detailed for replication, including sampling and assessment tools used (CHARLS data, CES-D scale for depression).
• The statistical analyses appear appropriate, utilizing regression methods and sensitivity analyses to ensure robustness in findings.
• The methodology does attempt to address potential biases by including various covariates and controlling for other factors influencing mental health.
• Research was conducted in an ethical manner, approved by the relevant institutional review board.
• The findings are presented clearly, with appropriate statistical results discussed, though incorporating more visual aids could enhance clarity.
• Results align well with stated objectives, showing significant associations between agricultural work, depressive symptoms, and life satisfaction.
• While the initial analyses are thorough, sensitivity analyses and confidence intervals could enhance the validity of the results.
• The authors provide evidence supporting their claims, referencing statistical findings effectively.
• The discussion includes comparisons with existing literature, although a more in-depth exploration of how findings differ from or confirm previous studies could strengthen arguments.
• The alternative interpretations of findings considered are not considered. The authors could expand on alternative interpretations of their findings for a more balanced discussion.
• Conclusions logically follow the data and analyses presented.
• Limitations are acknowledged, but a deeper discussion on their implications could enhance the conclusion.
• The study does suggest avenues for future research, particularly in improving life satisfaction among agricultural workers.
• The manuscript is well-organized, though some sections could benefit from more concise presentations.
• The writing is generally clear and accessible, suitable for readers with a general background in health research.

Reply: It is our honor to have your approval of our revised manuscript. We really appreciate your precious comments and advice. And we would continue our further study on Job diversities with mental health and try to figure out more reasonable explanation based on our current research, especially try to decline our limitations during collecting data and designing our study.

Thank you again for your highly speak of our work.

Best regards,

Yuan Yao Chen
