# Supplementary material for: Can working in agriculture have a favorable effect on depressive symptoms? Life satisfaction as a mediator
Source: PLoS One. 2025 Aug 13;20(8):e0325912. doi: 10.1371/journal.pone.0325912 (PMC12349095; doi:10.1371/journal.pone.0325912)
Supplement: Attachment — Submitted filename: Reply to reviewer3.docx [file pone.0325912.s005.docx]

Dear Reviewer3,

Thank you for your detailed comments and sincere help to help us improve our further research quality and enhance our narration of our limitations. We are sorry that due to the structure our original data set and observational study, it is hard to make deeper revision on the current study, however, we would continue to make our future research more complete under your guidance.

The study addresses a critical issue and Utilizing the CHARLS 2015 dataset (n=6,856) enhances statistical power and generalizability to China’s middle-aged and elderly population. The use of CES-D for depressive symptoms and SWLS for life satisfaction strengthens the reliability of the outcomes. Inclusion of demographic, behavioral, and social factors (e.g., age, gender, social activity) reduces confounding bias. Mediation analysis via Baron and Kenny’s approach and Sobel test appropriately tests the hypothesized pathways.
However, consider the following concern to address them Limits causal inference. The observed associations could reflect reverse causality (e.g., depressive individuals may self-select into agricultural work).
1. Excluding 11,270 participants due to missing values risks selection bias. Multiple imputation or sensitivity analyses are needed to validate findings.
2. Dichotomizing jobs into "agricultural" vs. "non-agricultural" ignores heterogeneity within agricultural work (e.g., subsistence vs. commercial farming). Clarify contradictions (e.g., job type’s non-significance in logistic regression vs. abstract’s emphasis on self-employed risk).
3. Reliance on one question ("How satisfied are you with your life?") may lack depth compared to multi-item scales like SWLS.
4. Life satisfaction mediated only 5.13% of the total effect, questioning its practical significance. Other mediators (e.g., income stability, social support) were overlooked.
5. Self-employed agricultural workers showed more protective factors, conflicting with literature on self-employment stressors. The rationale for this is underexplored.
6. The 2015 dataset may not reflect recent socioeconomic changes in rural China (e.g., digitalization, policy shifts).
7. Inconsistent table/figure numbering, (e.g., Fig. 3–7 not provided), and unclear reporting of propensity score matching methodology. Provide full details on propensity score matching (variables, method, balance diagnostics).

Highlight them in limitation and recommend them for future consideration.
1. Use longitudinal data to establish temporal relationships.
2. Apply multiple imputation for missing data and report sensitivity analyses.
3. Refine job type categorization (e.g., differentiate farming practices, land ownership).
4. Consider alternative regression models (e.g., Poisson) for common outcomes to avoid overestimating odds ratios.
5. Explore additional mediators (e.g., economic stability, pesticide exposure) to explain the weak mediation effect of life satisfaction.
6. Include all figures/tables referenced (e.g., correlation matrix, mediation model).
7. Address any ethical and temporal relevance related to mental health.

Authors’ Reply: Thank you for your kind comment! We have added several advice and comment into our Section Discussion and Limitations, which were highlighted in yellow in our revised manuscript. Moreover, we have checked and revised the miss cited table&figure in the manuscript, cited them in the right order and reapplied them into the system. The revised limitations were as follows:

“This study has several limitations. First, it is based on cross-sectional data from the 2015 China Health and Retirement Longitudinal Study (CHARLS), which limits the ability to establish a clear temporal sequence between agricultural employment and depressive symptoms. Consequently, reverse causality cannot be ruled out; for instance, individuals with depression may be more inclined to engage in agricultural work.Longitudinal designs are essential for understanding the temporal relationship between job type and mental health outcomes. Existing longitudinal investigations have illuminated that the depressive symptoms is not static; rather, it can fluctuate over time, which cross-sectional studies may not adequately represent. Longitudinal research highlights the dynamic relationship between job type and mental health, with studies showing that unstable employment can lead to increased depressive symptoms over time[77] and work-related stressors can exacerbate mental health issues[78]. Future research should employ longitudinal data to more accurately elucidate causal relationships between variables. Second, a substantial number of samples (n = 11,270) were excluded due to missing covariate data, which may introduce selection bias. Future studies should consider employing multiple imputation techniques to handle missing data and conduct sensitivity analyses to assess the robustness of the findings. Additionally, the classification of occupational types was simplistic, dividing them merely into "agricultural" and "non-agricultural," failing to capture the heterogeneity within agricultural work, such as subsistence versus commercial farming and land ownership differences. Future research should refine occupational classifications to more comprehensively understand the impact of occupational types on depressive symptoms. The study also employed logistic regression to analyze depressive symptoms, a common outcome variable, which may overestimate odds ratios. Future studies might consider alternative regression models, such as Poisson regression, to obtain more accurate estimates. Although this study found that life satisfaction had a mediating effect between agricultural work and depressive symptoms, the effect was weak, accounting for only 5.13% of the total effect. This may be due to the omission of other potential mediating variables, such as economic stability and pesticide exposure. Future research should explore additional mediators to more fully understand the mechanisms by which agricultural work influences depressive symptoms. Finally, the 2015 data used in this study may not capture recent socioeconomic changes in rural China, such as digital development and policy shifts. Additionally, mental health research involves sensitive information, necessitating careful ethical considerations to ensure participant privacy and informed consent during data collection and analysis. Future studies should focus on the temporal relevance of data and enhance ethical review and participant protection measures.”

Best regards,

Yuan Yao Chen
