# Supplementary material for: Can working in agriculture have a favorable effect on depressive symptoms? Life satisfaction as a mediator
Source: PLoS One. 2025 Aug 13;20(8):e0325912. doi: 10.1371/journal.pone.0325912 (PMC12349095; doi:10.1371/journal.pone.0325912)
Supplement: Attachment — Submitted filename: response.docx [file pone.0325912.s006.docx]

Please review your reference list to ensure that it is complete and correct. If you have cited papers that have been retracted, please include the rationale for doing so in the manuscript text, or remove these references and replace them with relevant current references. Any changes to the reference list should be mentioned in the rebuttal letter that accompanies your revised manuscript. If you need to cite a retracted article, indicate the article’s retracted status in the References list and also include a citation and full reference for the retraction notice.

Dear Editor,

Thank you for your kind reminder, we have searched and checked all the cited references, and made sure that all the list is complete, correct and no paper were retracted in present. Thank you for the kind reminder again!

Best Regards,

Yuan Yao Chen
