# Supplementary material for: Can working in agriculture have a favorable effect on depressive symptoms? Life satisfaction as a mediator
Source: PLoS One. 2025 Aug 13;20(8):e0325912. doi: 10.1371/journal.pone.0325912 (PMC12349095; doi:10.1371/journal.pone.0325912)
Supplement: Attachment — Submitted filename: Reply 5.19.docx [file pone.0325912.s007.docx]

Dear Editor,

We sincerely appreciate the editor’s meticulous review and constructive feedback. Below is our point-by-point responsethe issues raised, along with the revisions made to the manuscript:

I have carefully reviewed the manuscript and would like to share some editorial suggestions to enhance the clarity and professionalism of the text:

Inconsistent Spacing:

In several instances, there are missing spaces after periods and commas, e.g., "depressive symptoms.Life satisfaction" should be "depressive symptoms. Life satisfaction."

Authors’ reply：Thank you for your thoroughly check and review, all instances of missing or extra spacing have been corrected. For example, revised“depressive symptoms.Life satisfaction”to“depressive symptoms. Life satisfaction.” Adjusted spacing in phrases like “working in agriculture,” to ensure proper formatting, which were all highlighted in yellow.

Extra spaces were found between words in sentences like "working in agriculture," which should be corrected to "working in agriculture."

In many instances, reference numbers are directly attached to the last word, e.g., "study shows that depression is prevalent among older adults[12]" should be "study shows that depression is prevalent among older adults [12]."

Authors’ reply：Thank you for your thoroughly check and review, spaces have been added between text and reference numbers in all instances. We have made sure all reference numbers with spaces attached to the last word.

Improper Hyphenation and Spacing:

Some compound terms such as "self-employedagricultural workers" lack appropriate spacing. This should be revised to "self-employed agricultural workers."

Authors’ reply：Thank you for your thoroughly check and review, all compound terms have been standardized with appropriate spacing: Corrected “self-employedagricultural workers” to “self-employed agricultural workers.”The corrections were in yellow in the revised manuscript.

Extra Periods:

In certain parts of the manuscript, extra periods were identified, e.g., "data were analyzed.." This should be corrected to "data were analyzed."

Spacing Around Statistical Values:

In statistical reporting, there should be a space before and after inequality symbols, e.g., "p<0.001" should be "p < 0.001."

Authors’ reply：Thank you for your thoroughly check and review, redundant periods have been removed. Examples include: Revised “data were analyzed..” to “data were analyzed.”Checked for duplicated punctuation in tables, footnotes, and body text.

Inconsistent Use of Punctuation:

Some sections contain inconsistent use of commas and semicolons. Please ensure that list items are separated correctly and that sentences are punctuated properly.

Suggestions for Improvement:

I recommend a thorough proofreading to ensure consistency in spacing, punctuation, and formatting.

Utilizing tools such as Grammarly or the built-in proofing tools in Microsoft Word may assist in identifying minor errors.

Authors’ reply：Thank you for your thoroughly check and review, punctuation has been standardized: Corrected list separators (e.g., revised “factors such as work pressure \[5\], work hours \[6, 7\]” to ensure commas and semicolons align with journal guidelines). Adjusted sentence structures for clarity and consistency (e.g., removed misplaced semicolons in tables).

Best regards,

Yuan Yao Chen
